# The association between triglyceride glucose-body mass index and live birth outcomes in women with clinical pregnancy after frozen-thawed embryo transfer: a cohort study

**DOI:** 10.1186/s12884-026-08926-4

**Published:** 2026-03-12

**Authors:** Xiliang Wang, Pengshu Zou, Jing Zhou, Yuexin Yu

**Affiliations:** Department of Reproductive Medicine, General Hospital of Northern Theater Command, Shenyang, 110003 China

**Keywords:** Insulin resistance, Triglyceride glucose-body mass index, Live birth rate, In vitro fertilization, Frozen-thawed embryo transfer

## Abstract

**Background:**

Metabolic syndrome is common among infertile women and is closely related to insulin resistance (IR). Triglyceride glucose-body mass index (TyG BMI) is a newly explored indicator of IR. Currently, the association between TyG BMI and live birth outcomes has been studied in women with polycystic ovary syndrome (PCOS) undergoing in vitro fertilization (IVF), However, there is a lack of research on the general population of frozen-thawed embryo transfer (FET).

**Methods:**

This is a cohort study based on data from a reproductive center in China from January 2016 to December 2023. The association between TyG BMI and live birth outcomes in women with clinical pregnancy after FET was evaluated using generalized additive model(GAM) and threshold saturation effect analysis.

**Results:**

A total of 3,709 cycles resulted in clinical pregnancy after FET, with a mean age of 32.76 ± 3.99 years. Among these, 2860 cycles (77.11%) resulted in live births, with a mean age of 32.46 ± 3.81 years. After adjusting for confounding factors, TyG BMI was negatively correlated with live birth outcomes (OR = 0.994, 95% CI: 0.992, 0.996; *p* < 0.001). GAM analysis showed that the smooth term of TyG BMI was significant (*p* < 0.001), indicating a nonlinear relationship. Threshold effect analysis identified a breakpoint (K) of 176.849 (95% CI: 168.097, 186.702). When TyG BMI was below 176.849, The increase in the live birth rate was not significant, but when TyG BMI exceeded 176.849, for each additional unit, the chance of a live birth decreases by approximately 0.8% (OR = 0.992, *p* < 0.001).

**Conclusion:**

Our findings suggest that a higher TyG BMI is negatively associated with live birth outcomes in women with clinical pregnancy after FET. Further research is needed to validate these findings.

## Introduction

In 2023, the World Health Organization (WHO) reported that approximately one in six people worldwide experience infertility during their lifetime [1]. Metabolic syndrome is characterized by abdominal obesity, hypertension, hyperglycemia, and dyslipidemia, all of which can negatively impact female fertility [2]. Metabolic syndrome is highly prevalent among infertile women and is closely associated with insulin resistance (IR) [3, 4].

IR refers to a reduced response of the body to insulin [5]. During in vitro fertilization (IVF), IR may affect ovarian function, oocyte quality, and embryo development [6, 7]. It may also reduce the pregnancy success rate by impairing decidualization, reducing endometrial receptivity, and disrupting the embryo implantation process [8].

The triglyceride glucose-body mass index (TyG BMI) is an emerging surrogate marker for assessing the degree of IR [9]. It combines the triglyceride-glucose index (TyG) and body mass index (BMI), providing a more comprehensive reflection of an individual’s metabolic status. It outperforms other indicators, such as TyG and Homeostatic Model Assessment of Insulin Resistance (HOMA-IR) in predicting live birth rates in fresh embryo transfer cycles [10]. Several studies have explored the potential predictive role of TyG BMI in IVF outcomes. TyG BMI is negatively correlated with live birth rates in fresh embryo transfer cycles among patients with polycystic ovary syndrome (PCOS). and is a valuable predictor of adverse pregnancy outcomes [11].

Considering that the research on the relationship between TyG BMI and live birth outcomes in women with clinical pregnancy of frozen-thawed embryo transfer (FET) cycles is scarce, we carried out a cohort study to systematically bridge this epidemiological gap.

## Methods

### Study design and participants

This was a single-center retrospective cohort study conducted at the Reproductive Medicine Department of the Northern Theater General Hospital in Shenyang, Liaoning Province, China, from January 2016 to December 2023. The study included patients with clinical pregnancy resulting from frozen embryo transfer via IVF. The study was approved by the Reproductive Ethics Committee of the Northern Theater General Hospital (Approval No. 2025-2).

There are many factors that affect the live birth rate after FET, such as embryo quality, endometrial quality [12, 13]. At present, the quality of embryos is mainly judged based on their morphology, but this method is not very accurate [14]. The quality of the endometrium is mainly judged based on its thickness and morphology, but the influence of the endometrium on embryo implantation and development is not consistent as reported in the literature [15]. Therefore, this study selects the clinical pregnancy population after FET, which can largely eliminate the influence of embryo quality and endometrial quality.

The inclusion criteria for this study were as follows: (1) women with clinical pregnancy after FET. Clinical pregnancy is defined as the detection of a gestational sac by ultrasound four weeks after embryos transfer.

The exclusion criteria were as follows: (1) Missing basic information and biochemical data. (2) Ectopic pregnancy. (3) Triple pregnancy. (4) Termination of pregnancy for fetal anomalies. (5) Loss to follow-up.

**Fig. 1:**
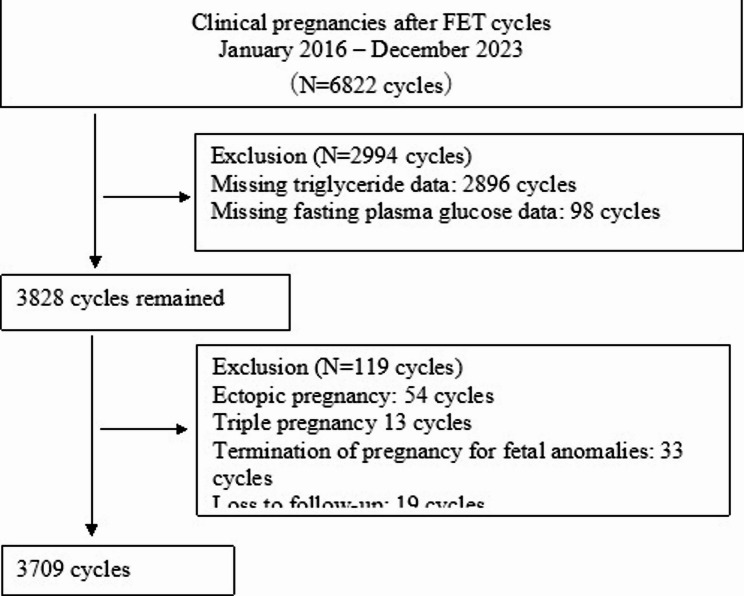
Flow chart of patients’ selection and exclusion. FET: frozen-thawed embryo transfer

After exclusions, 3,709 cycles remained. (Fig. 1)

### Ovarian stimulation protocol and oocyte retrieval

Ovarian stimulation is carried out using long protocol, antagonist protocol, or mild stimulation protocol based on the patient’s ovarian function. When ultrasound indicates that 1–2 dominant follicles have a diameter of ≥ 18 mm, 5,000 to 10,000 U of human chorionic gonadotropin (hCG, Ovitrelle, Merck Serono, Switzerland) is administered intramuscularly. 36 h later, follicular aspiration is performed via transvaginal ultrasound to collect oocytes.

### Embryo culture and embryo cryopreservation

The fertilization method, including IVF and Intracytoplasmic Sperm Injection (ICSI), was determined based on sperm quality and previous fertilization results. Post-fertilization embryos were cultured to the third day or the sixth day. Embryo cryopreservation was performed using the vitrification method (Kato, Japan), and the operation was carried out according to the instructions of the kit.

### Endometrium preparation and embryo transfer

Exogenous estradiol was used to establish an artificial menstrual cycle, and FET was carried out 3 or 5 days after the addition of progesterone.

### Exposure variables and definitions

PCOS was diagnosed using the Rotterdam Criteria, requiring at least two of the following: oligomenorrhea or anovulation, clinical or biochemical hyperandrogenemia, and polycystic ovaries on ultrasound, while excluding other causes of hyperandrogenemia and ovulatory dysfunction [16–18].

Biochemical blood tests and anthropometric measurements are carried out within one month before initiating the embryo transfer cycle. Basal hormonal profiling is conducted on menstrual cycle days 2–4. Systolic Blood Pressure (SBP) and Diastolic Blood Pressure (DBP) were measured using an electronic sphygmomanometer. The collected data included Fasting Plasma Glucose (FPG), Fasting insulin, Triglycerides (TG), Total Cholesterol (TC), High-Density Lipoprotein Cholesterol (HDL-C), Low-Density Lipoprotein Cholesterol (LDL-C), basal Follicle-stimulating Hormone (basal FSH), basal Luteinizing Hormone (basal LH), basal Prolactin (basal PRL), basal Estradiol (basal E2), basal Testosterone (basal T) and Anti-Müllerian Tubular Hormone (AMH). Biochemical indicators and basal hormonal profiling were measured after an overnight fast of > 8 h, between 7:30 AM and 9:30 AM. FPG was measured using the hexokinase method (Beckman Coulter). Fasting insulin, TG, TC, HDL-C, LDL-C, basal FSH, basal LH, basal PRL, basal E2, basal T, and AMH were assessed using electrochemiluminescence immunoassay (Roche, Germany).

The TyG BMI was calculated as follows [19].

$$\begin{aligned}\mathrm{TyG}\;\mathrm{BMI} =& \mathrm{Ln} \left[\mathrm{fasting}\;\mathrm{TG}\;\left(\mathrm{mg/dL}\right)\times\;\mathrm{FPG}\;\left(\mathrm{mg/dL}\right)/2\right]\\&\times\mathrm{BMI}\;\left(\mathrm{kg/m}^2\right)=\mathrm{Ln}\left[\mathrm{fasting}\;\mathrm{TG}\;\left(\mathrm{mmol/L}\right)\right.\\&\left.\times 88.57\times\mathrm{FPG}\;\left(\mathrm{mmol/L}\right)\times18/2\right]\times\mathrm{BMI}\;\left(\mathrm{kg/m}^2\right)\end{aligned}$$

### Outcome variables and definitions

The outcome variable was the live birth rate. Live birth was defined as the birth of a live infant at ≥ 28 weeks of gestation.

### Missing data handling

Observations with missing live birth outcome or primary exposure variables were excluded from the analysis. For covariates with < 5% missing data, no imputation or further processing was performed, as their impact on the analysis was considered. negligible. For variables with more than 5% missing values, multiple imputation was used to handle missing data.

### Statistical analysis

All analyses were performed using R (version 4.2; The R Foundation) and EmpowerStats (X&Y Solutions, Inc.). Continuous variables were expressed as mean ± standard deviation (normally distributed) or median (interquartile range) (non-normally distributed), while categorical variables were presented as frequencies (percentages). Group comparisons were conducted using one-way ANOVA (normally distributed data), Kruskal-Wallis test (non-normally distributed data), or chi-square/Fisher’s exact test (categorical variables). Multivariable regression analyses evaluated the association between TyG BMI and live birth rate, with results expressed as odds ratios (ORs) (logistic regression) and 95% confidence intervals (CIs). Three progressively adjusted models were constructed: (1) crude model (no adjustments); (2) minimally adjusted model (age); and (3) fully adjusted model (age, smoking status, systolic/diastolic blood pressure, PCOS status, diabetes, embryo transfer stage, and number of embryo transfers/implantations). Sensitivity analyses included stratification/interaction tests (adjusting for all covariates except stratification variables) and threshold saturation effect analysis to identify potential nonlinear relationships. Statistical significance was defined as two-sided *p* < 0.05 for all analyses.

## Results

### Baseline characteristics

A total of 3,709 participants were included in this study. The mean age of participants was 32.76 ± 3.99 years, the mean BMI was 23.65 ± 3.87 kg/m², and the mean TyG BMI was 200.19 ± 41.80. Among the participants, 2.56% reported a history of smoking. The proportion of primary infertility was 57.35%, and 31.49% were diagnosed with PCOS. Regarding the Number of embryo Implanted, 2,871 women had a single embryo implantation, while 838 had double embryo implantations. For delivery outcomes, 2,320 women experienced singleton deliveries and 540 experienced twin deliveries.

Across TyG BMI quartiles, there were clear and consistent trends: participants in the higher TyG BMI quartiles (Q4) were generally older, had greater body weight, and higher BMI (all *p* < 0.001). Moreover, increasing TyG BMI was associated with a progressively unfavorable metabolic profile, including higher fasting plasma glucose, fasting insulin, total cholesterol, triglycerides, and LDL-C levels, accompanied by lower HDL-C (all *p* < 0.001). Participants with higher TyG BMI also exhibited elevated systolic and diastolic blood pressure, a higher prevalence of PCOS, longer duration of infertility, and an increased proportion of secondary infertility (all *p* < 0.001 or *p* < 0.05). In contrast, no significant differences were found among the different TyG BMI quartile groups regarding the developmental stage of embryos transfer, the number of embryos transferred, or the number of embryos implanted.

Regarding pregnancy outcomes across TyG BMI quartiles, a notable gradient was observed. With increasing TyG BMI, the proportion of pregnancy loss—particularly early and late miscarriage—rose significantly (*p* < 0.001). The incidence of pregnancy loss in the highest TyG BMI quartile (Q4) was considerably higher compared with the lowest quartile (Q1). Similarly, the rate of women with no live births also increased across the TyG BMI quartiles, indicating a potential negative association between TyG BMI and live birth outcomes. (Table 1)

**Table 1:** Baseline characteristics of participants

Characteristics	Overall	TyG BMI quartile				
		Q1	Q2	Q3	Q4	*p* value
N	3709	928	927	927	927	
Age(years)	32.76 ± 3.99	32.25 ± 3.87	32.79 ± 4.15	32.98 ± 3.94	33.03 ± 3.96	< 0.001
Height(cm)	162.67 ± 4.84	163.16 ± 4.71	162.89 ± 4.97	162.33 ± 4.71	162.30 ± 4.91	< 0.001
Weight(kg)	62.62 ± 10.76	51.87 ± 4.57	58.45 ± 4.87	64.45 ± 5.27	75.71 ± 9.14	< 0.001
BMI(kg/m^2)	23.65 ± 3.87	19.47 ± 1.34	22.01 ± 1.23	24.43 ± 1.38	28.71 ± 2.89	< 0.001
Infertility duration(years)	3.00 (2.00–5.00)	3.00 (2.00–5.00)	3.00 (2.00–5.00)	3.00 (2.00–5.00)	4.00 (2.00–6.00)	< 0.001
FPG(mmol/L)	5.17 ± 1.32	4.94 ± 0.44	5.08 ± 1.56	5.17 ± 0.48	5.48 ± 2.00	< 0.001
Fasting insulin(pmol/L)	12.41 (7.85–22.84)	7.49 (5.24–11.50)	9.68 (6.66–15.25)	13.50 (9.63–21.36)	21.17 (14.57–34.36)	< 0.001
TC(mmol/L)	4.45 ± 0.89	4.24 ± 0.68	4.35 ± 0.76	4.50 ± 0.82	4.73 ± 1.14	< 0.001
TG(mmol/L)	1.06 (0.74–1.58)	0.68 (0.54–0.85)	0.90 (0.72–1.18)	1.24 (0.92–1.58)	1.84 (1.33–2.50)	< 0.001
HDL-C(mmol/L)	1.36 ± 0.44	1.55 ± 0.29	1.43 ± 0.47	1.29 ± 0.43	1.16 ± 0.46	< 0.001
LDL-C(mmol/L)	2.85 ± 0.79	2.59 ± 0.86	2.78 ± 0.68	2.96 ± 0.73	3.08 ± 0.79	< 0.001
SBP(mmHg)	117.29 ± 13.27	111.48 ± 10.27	114.44 ± 10.58	118.51 ± 12.25	124.74 ± 15.46	< 0.001
DBP(mmHg)	74.01 ± 9.88	69.60 ± 8.00	72.03 ± 8.71	74.58 ± 9.14	79.83 ± 10.47	< 0.001
Basal FSH(mIU/L)	5.93 (5.03–6.99)	6.27 (5.34–7.42)	6.09 (5.18–7.16)	5.82 (5.03–6.92)	5.53 (4.71–6.51)	< 0.001
Basal LH(mIU/L)	5.03 (3.60–7.06)	5.46 (4.12–7.16)	5.12 (3.75–6.99)	4.75 (3.34–6.45)	4.71 (3.23–7.37)	< 0.001
Basal PRL(mIU/L)	332.60 (246.10-445.85)	357.00 (273.20-466.80)	358.10 (261.35–461.40)	326.80 (245.10-439.80)	292.60 (218.50-413.40)	< 0.001
Basal E2(pg/L)	39.34 (29.28–52.64)	44.86 (33.59–61.10)	41.09 (30.14–53.35)	36.69 (27.55–47.33)	36.45 (27.34–47.78)	< 0.001
Basal T(ng/mL)	0.27 (0.18–0.37)	0.24 (0.17–0.33)	0.26 (0.17–0.35)	0.27 (0.18–0.37)	0.30 (0.21–0.44)	< 0.001
AMH(ng/mL)	3.27 (1.90–5.43)	3.06 (1.75–4.91)	3.02 (1.76–5.02)	3.27 (2.02–5.49)	3.80 (2.16–6.37)	< 0.001
Menarche(years)	13.72 ± 1.11	13.82 ± 1.04	13.75 ± 1.11	13.73 ± 1.15	13.59 ± 1.12	< 0.001
TyG-BMI	200.19 ± 41.80	153.67 ± 10.38	180.73 ± 7.60	208.05 ± 8.78	258.36 ± 27.52	< 0.001
Smoking						0.008
No	3614 (97.44%)	909 (97.95%)	909 (98.06%)	907 (97.84%)	889 (95.90%)	
Yes	95 (2.56%)	19 (2.05%)	18 (1.94%)	20 (2.16%)	38 (4.10%)	
Infertility type						0.017
Primary	2127 (57.35%)	571 (61.53%)	532 (57.39%)	516 (55.66%)	508 (54.80%)	
Secondary	1582 (42.65%)	357 (38.47%)	395 (42.61%)	411 (44.34%)	419 (45.20%)	
PCOS						< 0.001
No	2541 (68.51%)	759 (81.79%)	713 (76.91%)	624 (67.31%)	445 (48.00%)	
Yes	1168 (31.49%)	169 (18.21%)	214 (23.09%)	303 (32.69%)	482 (52.00%)	
Diabetes						< 0.001
No	3667 (98.89%)	928 (100.00%)	924 (99.78%)	922 (99.46%)	893 (96.33%)	
Yes	41 (1.11%)	0 (0.00%)	2 (0.22%)	5 (0.54%)	34 (3.67%)	
Etiology of infertility						< 0.001
Female factors	2439 (65.78%)	568 (61.21%)	588 (63.50%)	626 (67.53%)	657 (70.87%)	
Male factors	534 (14.40%)	171 (18.43%)	158 (17.06%)	120 (12.94%)	85 (9.17%)	
Combined factors	735 (19.82%)	189 (20.37%)	180 (19.44%)	181 (19.53%)	185 (19.96%)	
Fertilization method						0.065
IVF	1978 (53.33%)	469 (50.54%)	497 (53.61%)	525 (56.63%)	487 (52.54%)	
ICSI	1731 (46.67%)	459 (49.46%)	430 (46.39%)	402 (43.37%)	440 (47.46%)	
Number of embryo transferred						0.267
1	1292 (34.83%)	333 (35.88%)	316 (34.09%)	303 (32.69%)	340 (36.68%)	
2	2417 (65.17%)	595 (64.12%)	611 (65.91%)	624 (67.31%)	587 (63.32%)	
Developmental stage of embryo transferred						0.270
Cleavage stage	1221 (32.92%)	312 (33.62%)	286 (30.85%)	299 (32.25%)	324 (34.95%)	
Blastocyst stage	2488 (67.08%)	616 (66.38%)	641 (69.15%)	628 (67.75%)	603 (65.05%)	
Number of embryo implanted						0.775
1	2871 (77.41%)	716 (77.16%)	721 (77.78%)	708 (76.38%)	726 (78.32%)	
2	838 (22.59%)	212 (22.84%)	206 (22.22%)	219 (23.62%)	201 (21.68%)	
Pregnancy loss						< 0.001
No	2860 (77.11%)	756 (81.47%)	749 (80.80%)	725 (78.21%)	630 (67.96%)	
Early miscarriage	678 (18.28%)	152 (16.38%)	153 (16.50%)	161 (17.37%)	212 (22.87%)	
Late miscarriage	171 (4.61%)	20 (2.16%)	25 (2.70%)	41 (4.42%)	85 (9.17%)	
Outcome						< 0.001
Non-live birth	849 (22.89%)	172 (18.53%)	178 (19.20%)	202 (21.79%)	297 (32.04%)	
Live birth	2860 (77.11%)	756 (81.47%)	749 (80.80%)	725 (78.21%)	630 (67.96%)	
Number of deliveries						0.318
1	2320 (81.12%)	602 (79.63%)	604 (80.64%)	588 (81.10%)	526 (83.49%)	
2	540 (18.88%)	154 (20.37%)	145 (19.36%)	137 (18.90%)	104 (16.51%)	

Data are expressed as median (interquartile range) for non-normally distributed continuous variables

Data are expressed as mean + SD for normally distributed continuous variables

Categorical variables were expressed in frequency or as a percentage

*Abbreviations*: *BMI* Body Mass Index, *SBP* Systolic blood pressure, *DBP* Diastolic blood pressure,*FPG* Fasting Plasma Glucose, *TC* Total Cholesterol, *TG* Triglycerides, *HDL-C* High Density Lipoprotein Cholesterol, *LDL-C* Low Density Lipoprotein Cholesterol, *TyG BMI* Triglyceride glucose-body Mass Index, *PCOS* Polycystic Ovary Syndrome, *basal FSH* basal Follicle-stimulating Hormone, *basal LH* basal Luteinizing Hormone, *basal PRL* basal Prolactin, *basal E2* basal Estradio, *basal T* basal Testosterone, *AMH* Anti-Müllerian Tubular Hormone, *IVF* In vitro fertilization, *ICSI* Intracytoplasmic Sperm Injection

### Association between TyG BMI and Live birth rate

The study examined the association between TyG BMI and live birth outcomes in women with clinical pregnancy undergoing FET cycles. As shown in Table 2, higher TyG BMI was consistently associated with reduced odds of live birth. Specifically, in the crude model, each unit increase in TyG BMI was linked to a significantly decreased odds of live birth (OR 0.993, 95% CI: 0.991, 0.994, *p* < 0.001). This inverse association remained robust after adjustment for age (minimally adjusted model: OR 0.993, 95% CI: 0.991, 0.995, *p* < 0.001) and further adjustment for age, smoking, blood pressure, PCOS, embryo development stage, number of embryos transferred, number of embryos implanted, and diabetes (fully adjusted model: OR 0.994, 95% CI: 0.992, 0.996, *p* < 0.001).

When TyG BMI was analyzed as quartiles, women in the highest TyG BMI quartile (Q4) had a significantly lower likelihood of live birth compared to those in the lowest quartile (Q1), with an adjusted odds ratio of 0.591 (95% CI: 0.462, 0.756, *p* < 0.001) in the fully adjusted model. (Table 2)

**Table 2:** Associations between TyG BMI and live birth in women with clinical pregnancy of FET cycles

Variable	Crude model (OR, 95%CI, *p*)	Minimally adjusted model (OR, 95%CI, *p*)	Fully adjusted model (OR, 95%CI, *p*)
TyG BMI	0.993 (0.991, 0.994) <0.001	0.993 (0.991, 0.995) < 0.001	0.994 (0.992, 0.996) < 0.001
TyG BMI quartile			
Q1	Ref	Ref	Ref
Q2	0.957 (0.759, 1.208) 0.713	1.004 (0.794, 1.270) 0.972	1.042 (0.821, 1.324) 0.732
Q3	0.817 (0.650, 1.025) 0.080	0.865 (0.687, 1.088) 0.215	0.930 (0.732, 1.181) 0.551
Q4	0.483 (0.389, 0.599) < 0.001	0.508 (0.408, 0.631) < 0.001	0.591 (0.462, 0.756) < 0.001

Crude model adjusts for: None

Minimally adjusted model adjusts for: Age(years)

Fully adjusted model adjusts for: Age(years); Smoking; SBP(mmHg); DBP(mmHg); PCOS; Diabetes; Developmental stage of embryo transferred; Number of embryo transferred; Number of embryo implanted

*Abbreviations*: *CI* Confidence interval, *OR* Odds ratio, *SBP* Systolic blood pressure, *DBP* Diastolic blood pressure, *PCOS* Polycystic Ovary Syndrome

### Subgroup analyses

Stratified analysis revealed that higher TyG-BMI was significantly associated with reduced live birth rates in several subgroups. Specifically, this inverse association was observed among women aged ≤ 35 years (OR = 0.993, 95% CI: 0.991, 0.996, *p* < 0.001), non-smokers (OR = 0.994, 95% CI: 0.992, 0.996, *p* < 0.001), women without PCOS (OR = 0.996, 95% CI: 0.994, 0.999, *p* = 0.010), and women with PCOS (OR = 0.992, 95% CI: 0.988, 0.995, *p* < 0.001). Similar results were found for both cleavage-stage (OR = 0.993, 95% CI: 0.990, 0.997, *p* < 0.001) and blastocyst-stage embryo transfer (OR = 0.995, 95% CI: 0.992, 0.997, *p* < 0.001). Moreover, the negative association remained significant regardless of whether one (OR = 0.996, 95% CI: 0.992, 0.999, *p* = 0.017) or two embryos were transferred (OR = 0.993, 95% CI: 0.991, 0.996, *p* < 0.001), as well as in cases with one (OR = 0.995, 95% CI: 0.993, 0.997, *p* < 0.001) or two embryos implanted (OR = 0.990, 95% CI: 0.985, 0.995, *p* < 0.001). Sensitivity analyses indicated results in each stratum consistently mirrored the overall findings. (Table 3)

**Table 3:** Subgroup analyses of the associations between TyG BMI and Live birth in women with clinical pregnancy of FET cycles

TyG BMI	*N*	OR (95%CI)	*p* value	*p* interaction
Age(years)				0.334
≤ 35	2808	0.993 (0.991, 0.996)	< 0.001	
36–40	767	0.996 (0.992, 1.001)	0.126	
>40	134	0.999 (0.988, 1.011)	0.892	
Smoking				0.714
No	3614	0.994 (0.992, 0.996)	< 0.001	
Yes	95	0.998 (0.985, 1.010)	0.706	
PCOS				0.004
No	2541	0.996 (0.994, 0.999)	0.010	
Yes	1168	0.992 (0.988, 0.995)	< 0.001	
Diabetes				0.528
No	3667	0.994 (0.992, 0.996)	< 0.001	
Yes	41	1.006 (0.982, 1.031)	0.612	
Developmental stage of embryo transfer				0.305
Cleavage stage	1221	0.993 (0.990, 0.997)	< 0.001	
Blastocyst stage	2488	0.995 (0.992, 0.997)	< 0.001	
Number of embryo transferred				0.383
1	1292	0.996 (0.992, 0.999)	0.017	
2	2417	0.993 (0.991, 0.996)	< 0.001	
Number of embryo Implanted				0.066
1	2871	0.995 (0.993, 0.997)	< 0.001	
2	838	0.990 (0.985, 0.995)	< 0.001	

For age adjusted for: Smoking; SBP(mmHg); DBP(mmHg); PCOS; Diabetes; Developmental stage of embryo transfer; Number of embryo transferred; Number of embryo Implanted

For Smoking adjusted for: Age(years); SBP(mmHg); DBP(mmHg); PCOS; Diabetes; Developmental stage of embryo transfer; Number of embryo transferred; Number of embryo Implanted

For PCOS adjusted for: Age(years); Smoking; SBP(mmHg); DBP(mmHg); Diabetes; Developmental stage of embryo transfer; Number of embryo transferred; Number of embryo Implanted

For Diabetes adjusted for: Age(years); Smoking; SBP(mmHg); DBP(mmHg); PCOS; Developmental stage of embryo transfer; Number of embryo transferred; Number of embryo Implanted

For Developmental stage of embryo transfer adjusted for: Age(years); Smoking; SBP(mmHg); DBP(mmHg); PCOS; Diabetes; Number of embryo transferred; Number of embryo Implanted

For Number of embryo transferred adjusted for: Age(years); Smoking; SBP(mmHg); DBP(mmHg); PCOS; Diabetes; Developmental stage of embryo transfer; Number of embryo Implanted

For Number of embryo Implanted adjusted for: Age(years); Smoking; SBP(mmHg); DBP(mmHg); PCOS; Diabetes; Developmental stage of embryo transfer; Number of embryo transferred

*Abbreviations*: *CI* Confidence interval, *OR* Odds ratio, *SBP* Systolic blood pressure, *DBP* Diastolic blood pressure, *PCOS* Polycystic Ovary Syndrome, *IVF* In Vitro Fertilization, *ICSI* Intracytoplasmic Sperm Injection

### Smooth curve fitting analysis

To further explore the relationship between TyG-BMI and live birth rate among women with clinical pregnancies, we employed generalized additive models (GAM) with smooth curve fitting. After adjusting for potential confounding variables including age, smoking status, systolic and diastolic blood pressure, number of embryos implanted, presence of PCOS, diabetes, embryo developmental stage, and number of embryos transferred, the model showed that TyG-BMI exhibits a significant non-linear association with live birth rate (smooth term for TyG-BMI: edf = 3.134, χ² = 35.53, *p* < 0.001). As TyG-BMI increased, the probability of live birth demonstrated a downward trend, especially in higher TyG-BMI ranges.

**Fig. 2:**
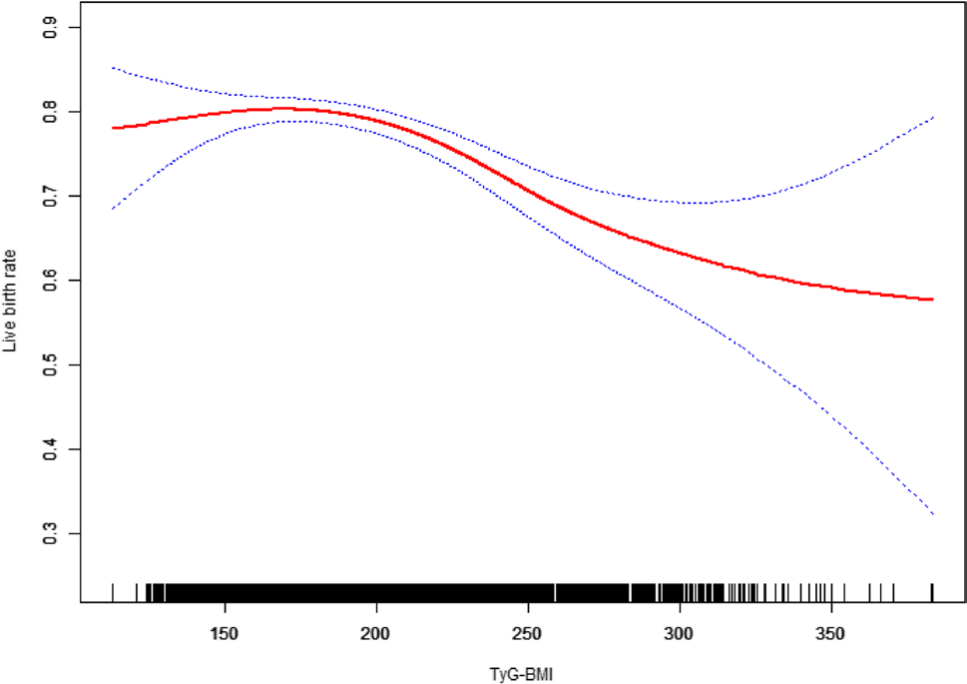
Association between TyG BMI and Live birth rate. Adjusted for Age(years); Smoking; SBP(mmHg); DBP(mmHg); PCOS; diabetes; Developmental stage of embryo transfer; Number of embryo transferred; Number of embryo Implanted. Abbreviations: SBP, Systolic blood pressure; DBP, Diastolic blood pressure; PCOS, Polycystic Ovary Syndrome

Among the covariates, advanced maternal age (OR = 0.92, 95% CI: 0.91, 0.94, *p* < 0.001), presence of PCOS (OR = 0.79, 95% CI: 0.66, 0.95, *p* = 0.012), and greater number of embryos transferred (OR = 0.65, 95% CI: 0.54, 0.79, *p* < 0.001) were all significantly associated with reduced odds of live birth. In contrast, a higher number of embryos implanted was associated with increased likelihood of live birth (OR = 2.40, 95% CI: 1.91, 3.02, *p* < 0.001). Other factors such as smoking, blood pressure, diabetes, and embryo developmental stage were not significant predictors. (Fig. 2)

### Threshold effect analysis

This study further evaluated the threshold effect of TyG BMI on live birth outcomes in women undergoing frozen embryo transfer (FET) cycles. Model I suggested a significant linear association, with an odds ratio (OR) of 0.994 (95% CI: 0.992, 0.996, *p* < 0.001), indicating that higher TyG BMI is associated with decreased odds of live birth. In Model II, a threshold (breakpoint) was identified at 176.849 (95% CI:168.097, 186.702). Below this breakpoint, TyG BMI was not significantly associated with live birth (OR = 1.006, 95% CI: 0.998, 1.015, *p* = 0.136). However, when TyG BMI exceeded the threshold, it was significantly and negatively associated with live birth outcomes (OR = 0.992, 95% CI: 0.990, 0.995, *p* < 0.001). The log-likelihood ratio test yielded a value of 0.004, confirming a statistically significant improvement with the threshold model and supporting the existence of a non-linear (threshold) effect between TyG BMI and live birth rate. These results were adjusted for age, smoking, systolic and diastolic blood pressure, PCOS, diabetes, embryo developmental stage, number of embryos transferred, and Number of embryo Implanted. (Table 4)

**Table 4:** Threshold effect analysis of TyG BMI and live birth rate

Live birth	OR	95% CI	*p* value
Model I			
Linear Effect	0.994	0.992, 0.996	< 0.001
Model II			
Breakpoint (K)	176.849	168.097, 186.702	
Segment Effect 1 (< K)	1.006	0.998, 1.015	0.136
Segment Effect 2 (> K)	0.992	0.990, 0.995	< 0.001
Log-Likelihood Ratio Test			0.004

Adjusted for Age(years); Smoking; SBP(mmHg); DBP(mmHg); PCOS; diabetes; Developmental stage of embryo transferred; Number of embryo transferred; Number of embryo Implanted

*Abbreviations*: *CI* Confidence interval, *SBP* Systolic blood pressure, *DBP* Diastolic blood pressure, *PCOS* Polycystic Ovary Syndrome

## Discussion

This study analyzes data from 3,709 women who achieved clinical pregnancy after undergoing FET, of whom 2,820 had a live birth. We find that a higher TyG BMI is negatively associated with live birth outcomes. Stratified analyses accounting for covariates demonstrated consistent results, showing reduced live birth associated with elevated TyG BMI in all subgroups. Notably, analysis using a GAM revealed a significant curvilinear relationship between increased TyG BMI and live birth rates, suggesting that within a certain range, elevated TyG BMI is associated with decreased live birth rates. Furthermore, a threshold effect was identified, and the breakpoint was 176.849 (95% CI:168.097, 186.702), indicating that exceeding a specific TyG BMI value significantly lowers the likelihood of live birth.

The hyperinsulinemic-euglycemic clamp technique is widely acknowledged as the gold standard for assessing IR and measuring insulin sensitivity [20]. However, its invasive, labor-intensive, costly, and technically demanding nature limits its application in large-scale clinical and epidemiological studies. Consequently, in recent years, alternative, simpler methods for evaluating IR have received growing attention in clinical research.

In 2016, Professor Er and colleagues introduced the TyG BMI index, which combines the TyG index and BMI [9]. A study found that TyG BMI is a more accurate indicator of IR compared to TyG alone [21]. Research has shown that the TyG BMI index outperforms the TyG index alone in identifying non-alcoholic fatty liver disease (NAFLD) in non-obese patients [22, 23]. Recent studies have demonstrated that the TyG BMI index is superior to the TyG index in accurately detecting metabolic syndrome in patients with PCOS [3]. This finding suggests that the TyG BMI index can serve as a comprehensive indicator of overall health issues. Shenghao Wu [10] reported that different IR surrogate markers (such as TyG BMI, TyG, and HOMA-IR) were negatively correlated with live birth rates, and this relationship was significant in fresh embryo transfer (ET) cycles but not in frozen-thawed embryo transfer (FET) cycles. However, the sample size in their study was limited (only 409 frozen-thawed embryo transfer cycles), and the population studied was restricted to women with PCOS. By comparison, our study involved a substantially larger cohort and included subjects from the general population undergoing FET. Our study superficially indicates a significant negative correlation between TyG BMI and live birth rate in women with clinical pregnancy after FET, with a threshold effect relationship.

Polycystic ovary syndrome (PCOS) is an endocrine and metabolic disorder characterized by imbalances of multiple hormones that reflect the clinical manifestations of hyperandrogenism and affects 5%–10% of women of childbearing age [24, 25]. It is believed that IR and obesity play prevalent roles in causing PCOS, and PCOS women show an higher increased comorbidities of IR, including obesity, dyslipidemia, hypertension than healthy women [26–28]. Existing studies have documented the association between IR and fertility outcomes in women with PCOS [29–32]. In non-PCOS patients, IR also showed a positive association with the total follicle count after controlled ovarian hyperstimulation(COH) [33] and can serve as a predictive factor for ovarian hyperstimulation syndrome (OHSS) [34]. A studiy have investigated the impact of IR on endocrine, metabolic, and reproductive outcomes in non-PCOS women undergoing assisted reproductive technology (ART). The results showed that 12.0% of the women were diagnosed with IR, and the women with IR had significantly higher FPG, LDL-C, and TG levels, along with significantly lower HDL-C levels. However, IR was not associated with clinical pregnancy, live birth, or miscarriage rates. It should be noted that the study used HOMA-IR, which does not account for the influence of blood lipids and BMI [35]. And anothor study also indicated that IR may adversely affect IVF outcomes in lean/non-obese women without PCOS [36]. Evidence also suggests that among non-PCOS women, increased HOMA-IR was positively associated with late miscarriage [37].

IR impairs female fertility through multiple interrelated mechanisms. It induces hyperinsulinemia, leading to excess androgen production, which disrupts follicular development and ovulation, particularly in women with PCOS [38, 39]. Elevated insulin levels can directly cause ovarian dysfunction, reduce oocyte maturation, fertilization rates, and embryo quality [31]. Additionally, IR compromises endometrial receptivity [40]. Furthermore, chronic low-grade inflammation and increased oxidative stress often accompany IR, collectively impairing normal ovarian and uterine function and further reducing fertility [41].

IR impairs fertility through several interconnected molecular pathways. It disrupts normal insulin signaling by undermining the activity of the insulin receptor and its downstream molecules (such as IRS-1, PI3K, and Akt), leading to deficient glucose uptake and altered energy metabolism in reproductive tissues [42]. This metabolic disturbance, combined with insulin-induced upregulation of steroidogenic enzymes like CYP17A1 [43], results in excessive androgen production, which in turn hampers follicular development and ovulation. Furthermore, IR activates inflammatory pathways (such as NF-κB) and increases pro-inflammatory cytokines (e.g., TNF-α, IL-6), contributing to chronic inflammation and oxidative stress that further compromise the ovarian and endometrial environment [44]. In addition, reduced expression of insulin-like growth factors and integrins in the endometrium lowers endometrial receptivity, while dysregulation of apoptotic and cell cycle genes (such as Bcl-2 and Bax) impairs granulosa cell and oocyte survival and maturation [45]. Altogether, these molecular abnormalities converge to negatively affect female reproductive function and fertility outcomes.

A growing body of evidence suggests that improving insulin resistance—whether through lifestyle modifications, such as diet and exercise, or pharmacological interventions—can enhance reproductive outcomes in women with IR and in the broader population undergoing fertility treatments. Interventions aimed at increasing insulin sensitivity, including the use of insulin-sensitizing agents [46] and regular physical activity [47], have been shown to improve ovulation and pregnancy rates. Therefore, for patients with higher TyG BMI, implementing targeted strategies to manage obesity and metabolic syndrome may significantly improve the success rates of assisted reproductive technologies such as IVF.

This study has the following limitations. (1) The missing triglyceride data of 2896 cycles in 6822 cycles of women with clinical pregnancy after FET are significant. This high level of missing data may affect the accuracy and reliability of the results. Data loss can lead to sample bias, which in turn may influence the interpretation and generalizability of the findings. (2) While the TyG-BMI–live birth association may retain residual confounding due to heterogeneous/incomplete complication data, this primarily reflects inconsistent diagnostic documentation of pregnancy complications (e.g., gestational diabetes, hypertensive disorders) across the multiple antenatal care providers serving our geographically dispersed population. (3) There may be other influencing factors that were not adjusted due to the lack of collected data, for example, the diagnosis and incidence of hyperlipidemia, medication information, physical therapy, dietary interventions, etc.(4) The retrospective design of this study may introduce recall bias. To enhance the robustness of findings, future studies should adopt prospective cohort designs with objective biomarker measurements (e.g., genomic data) for multi-modal validation. (5) The single region sampling in this study may limit the external validity of the findings. Subsequent research should incorporate more diverse multi-ethnic/racial populations to improve the generalizability of conclusions.

## Conclusion

Understanding the complex relationship between TyG BMI and fertility outcomes highlights the importance of developing personalized treatment strategies aimed at improving IR. By integrating lifestyle modifications, dietary adjustments, and medical interventions, healthcare providers can create more effective and individualized plans for individuals facing fertility challenges related to IR. The focus on emerging markers such as TyG BMI can guide further research and clinical practice, promoting advancements in reproductive health.

## Data Availability

Data set used in this study will be available from the corresponding author on reasonable request.

## Abbreviations

ART: Assisted reproductive technology
BMI: Body mass index
CI: Confidence interval
COH: Controlled ovarian hyperstimulation
ET: Embryo transfer
FET: Frozen-thawed embryo transfer
GAM: Generalized Additive Model
HDL-C: High density lipoprotein cholesterol
IR: Insulin resistance
IVF: In vitro fertilization
ICSI: Intracytoplasmic Sperm Injection
LDL-C: Low density Lipoprotein cholesterol
NAFLD: Non-alcoholic fatty liver disease
OHSS: Ovarian hyperstimulation syndrome
OR: Odds ratio
PCOS: Polycystic ovary syndrome
TG: Triglyceride
TyG: Triglyceride-glucose index
TyG BMI: Triglyceride glucose-body Mass Index
WHO: World Health Organization
